# Effects of Dietary Sterol-Rich Yeast Hydrolysate on Serum Lipid Indices, Intestinal Morphology, Digestive Enzyme Activities, Barrier-Related Gene Expression, and Cecal Microbiota in Yellow-Feathered Broiler Breeder Hens

**DOI:** 10.3390/ani16162620

**Published:** 2026-08-21

**Authors:** Yining Zhang, Zhifang Shi, Mengyun Li, Haoran Si, Bo Fan, Xuanyang Li, Yongzhen Li, Xiaotong Wang, Jian Liu, Lei Xi

**Affiliations:** 1 College of Animal Science, Xinjiang Agricultural University, No. 311 Nongda East Road, Urumqi 830052, China; 2 College of Animal Science and Technology, Henan University of Animal Husbandry and Economy, No. 6 Longzihu North Road, Zhengzhou 450046, China; 3 Henan Provincial Engineering Research Center for Healthy Livestock and Poultry Farming and Intelligent Equipment, No. 6 Longzihu North Road, Zhengzhou 450046, China

**Keywords:** sterol-rich yeast hydrolysate, yellow-feathered broiler breeder hen, serum lipid, intestinal morphology, digestive enzyme, barrier-related gene expression, cecal microbiota

## Abstract

This study evaluated dietary sterol-rich yeast hydrolysate (SYH) at 0, 0.2, 0.5, and 0.8 g/kg in 720 yellow-feathered broiler breeder hens for 6 weeks. The tested SYH, supplied as a powder, contained 9.24% mannan, 3.1% total sterols, 2.0% amino nitrogen, and 3.64 g/kg nucleotides. Serum lipoproteins, intestinal morphology, digestive enzyme activities, barrier-related gene expression, and cecal microbiota were assessed. Low-density lipoprotein cholesterol was higher in J-C than in Control, J-A, and J-B, whereas high-density lipoprotein cholesterol did not differ among treatments. Duodenal lipase showed a significant overall treatment effect, but no Dunn–Holm pairwise comparison remained significant; ileal lipase was higher in J-C than in J-A. For barrier-related transcripts, J-A had higher ZO-1 than the Control and J-B and higher occludin and claudin-1 than all other groups, whereas MUC2 was higher in J-A and J-C than in the Control. These transcript changes are consistent with the modulation of molecular components associated with intestinal barrier integrity, but they do not by themselves demonstrate improved barrier function. Intestinal morphology showed selective, segment-specific differences rather than a consistent dose-related pattern. Alpha diversity did not differ, whereas Bray–Curtis PERMANOVA detected an overall difference among treatments (*p* = 0.017) despite overlapping PCoA distributions. Pairwise differential-abundance analysis using metagenomeSeq identified six family- or genus-level differences after false-discovery-rate correction, but these were specific to individual pairwise comparisons and showed no monotonic dose pattern. No exploratory microbiota–phenotype correlation remained significant after multiple-testing correction. Overall, SYH elicited inclusion-level- and trait-specific responses rather than a coordinated improvement across endpoints; therefore, the present data do not identify a single optimal inclusion level.

## Simple Summary

Sterol-rich yeast hydrolysate is a yeast-derived feed additive containing mannans, sterols, amino nitrogen, and nucleotides. We evaluated whether its inclusion at 0.2, 0.5, or 0.8 g/kg diet produced broad or trait-specific responses in serum lipoproteins, intestinal morphology, digestive enzyme activities, barrier-related gene expression, and cecal microbiota of yellow-feathered broiler breeder hens. Responses were selective rather than uniform. Low-density lipoprotein cholesterol was higher at 0.8 g/kg than in the other treatments, whereas high-density lipoprotein cholesterol did not differ. Digestive-enzyme responses were limited: duodenal lipase showed an overall treatment effect without a significant adjusted pairwise contrast, and ileal lipase was higher at 0.8 than at 0.2 g/kg. Barrier-related transcripts also showed endpoint-specific differences. Intestinal morphology varied selectively across segments, microbial alpha diversity remained stable, and six bacterial taxa differed in specific pairwise comparisons without a consistent dose pattern. No microbiota–phenotype correlation remained significant after multiple-testing correction. Thus, the additive altered selected endpoints, but the nonuniform response pattern does not support a single optimal inclusion level or a general improvement in intestinal health.

## 1. Introduction

The intestinal tract supports nutrient digestion and absorption while also providing a physical and immunological barrier and hosting a complex microbial community. In broiler breeder hens, maintaining these functions during the late laying period is relevant because digestive and metabolic status may become less stable with advancing age. Intestinal morphology, digestive enzyme activity, barrier-related gene expression, and cecal microbiota therefore provide complementary information on intestinal status. Yeast hydrolysates have been reported to affect intestinal morphology, barrier-associated responses, and immune or inflammatory traits in chickens, although the magnitude and direction of the responses have varied among products and experimental settings [1,2].

Yeast-derived feed additives are compositionally heterogeneous. Depending on the raw material and processing method, they may contain soluble cell-wall polysaccharides, peptides and amino nitrogen, nucleotides, sterols, and other intracellular fractions. Studies of yeast culture, yeast nucleotides, yeast cell walls, and yeast hydrolysates have reported changes in digestive enzyme activities, tight-junction-related endpoints, intestinal morphology, and microbial composition in chickens [3,4,5,6,7]. In laying hens, brewer’s yeast hydrolysate and yeast autolysate have produced variable responses in nutrient digestibility, egg traits, excreta microflora, and egg cholesterol [8,9]. This heterogeneity indicates that findings from one yeast-derived product or inclusion level cannot be directly generalized to another.

The sterol-rich yeast hydrolysate (SYH) evaluated in the present study differs from a purified sterol preparation because it contains mannans, sterols, amino nitrogen, nucleotides, and potentially other yeast-derived constituents. Dietary phytosterols or phytosterol esters have been associated with changes in serum lipids, intestinal morphology, antioxidant capacity, and hepatic metabolism in broilers or laying hens [10,11], whereas the productive response to yeast extract in aged laying hens has also been context-dependent [12]. More broadly, a recent review identifies mannans, β-glucans, nucleotides, and other intracellular yeast fractions as components that may contribute to the biological responses observed with hydrolyzed-yeast and yeast-culture products [13]. Experimental studies in broilers and laying or breeder hens have reported effects on mucosal morphology, digestive or immune indices, egg-related outcomes, and intestinal microbial profiles, but the magnitude and direction of response vary with product, dose, age, stressor, and production phase [14,15,16,17,18,19,20,21]. Because chicken intestinal communities are also sensitive to host, environmental, sampling, and analytical factors, microbiota responses should be interpreted within that broader context [22,23,24,25]. However, evidence is limited regarding whether a sterol-rich multicomponent yeast hydrolysate affects serum lipoproteins and selected intestinal endpoints in yellow-feathered broiler breeder hens, and whether any response is accompanied by a broad or selective change in the cecal bacterial community.

Accordingly, this study evaluated dietary supplementation with 0.2, 0.5, or 0.8 g SYH/kg diet in 55- to 61-week-old yellow-feathered broiler breeder hens. Serum high-density and low-density lipoprotein cholesterol, small-intestinal morphology and digestive enzyme activities, intestinal barrier-related gene expression, and cecal bacterial communities were assessed. We hypothesized that SYH would alter selected serum lipid and intestinal traits and that these responses might be accompanied by changes in cecal microbial composition. Because the product contains several bioactive fractions and the inclusion levels were not assumed to produce a linear response, the analysis emphasized whether effects were broad and dose-consistent or restricted to specific endpoints and pairwise treatment contrasts.

## 2. Materials and Methods

### 2.1. Additive, Animals, and Management

The sterol-rich yeast hydrolysate (SYH) used in this study was supplied by Hebei Ronghe Yeast Co., Ltd (Xingtai, China). as a powder product. A third-party laboratory tested a product sample from the same batch (report no. NZZ20231001695-2; submitted sample name: yeast sterol). Mannan, total sterol, amino nitrogen, and nucleotide contents were 9.24%, 3100 mg/100 g, 2.0 g/100 g, and 3.64 g/kg, respectively.

The experiment was conducted at a commercial broiler breeder farm in Henan Province, China. A total of 720 healthy 55-week-old yellow-feathered broiler breeder hens (SASSO SA51A; mean body weight, 2135.00 g; mean laying rate, 74.31%) were randomly allocated to four treatments. Each treatment comprised six replicates, with 30 hens per replicate. Each replicate consisted of five cages, with six hens per cage. Each cage measured 60 cm × 60 cm (length × width), with a front height of 50 cm and a rear height of 40 cm, providing 0.36 m^2^ of floor area and a stocking density of 16.7 hens/m^2^ (600 cm^2^/hen). Feed and water were provided ad libitum. Temperature (22–26 °C), relative humidity (50–70%), and photoperiod (15–16 h/day) were maintained as consistently as practicable among groups. The house was naturally ventilated, routine vaccination followed the farm program, and manure was removed regularly. The experimental period was 6 weeks (55–61 weeks of age).

### 2.2. Experimental Design

The study was approved by the Animal Ethics Committee of Henan University of Animal Husbandry and Economy (approval no. HNUAHE ER2607D601). The four treatments were the basal diet without supplemental SYH (Control) and the basal diet supplemented with 0.2 (J-A), 0.5 (J-B), or 0.8 g SYH/kg diet (J-C). The three supplementation levels were selected based on the manufacturer’s recommended product inclusion range and were intended to represent low, intermediate, and high inclusion levels within that range. SYH was top-dressed and did not replace other ingredients. Based on the guaranteed total sterol content, the additive theoretically supplied 6.2, 15.5, and 24.8 mg total sterols/kg diet in J-A, J-B, and J-C, respectively. These calculated sterol contributions are in or below the lower portion of ranges previously tested with purified phytosterols or phytosterol esters in poultry (10–80 mg/kg diet in broilers and 10–40 mg/kg diet in laying hens [10,11]); however, direct dose equivalence should not be assumed because SYH is a multicomponent hydrolysate rather than a purified sterol preparation. Because the maximum inclusion level was 0.08%, its contribution to dietary energy and protein was considered negligible, and no additional isoenergetic or isonitrogenous adjustment was made. A 1-week adaptation period preceded the 6-week experimental period. The basal diet was formulated according to the nutrient requirements used by the farm. Crude protein, crude fat, and calcium were determined according to GB/T 6432-2018 [26], GB/T 6433-2006 [27], and GB/T 6436-2018 [28], respectively; metabolizable energy and available phosphorus were calculated using the Chinese Feed Composition and Nutritive Value Tables (2021, 32nd edition) [29]. The ingredient composition and nutrient levels of the basal diet are presented in Appendix A (Table A1).

Each 30-hen replicate, comprising five physical cages with six hens per cage, was treated as one experimental unit. For serum lipid indices, digestive enzyme activities, and barrier-related gene expression, three hens were sampled from each replicate; measurements were obtained separately for each hen and then averaged to generate one replicate-level observation. For intestinal histomorphology, one designated sampled hen per replicate contributed one section per intestinal segment, and the three paired villus-crypt units measured within that section were averaged to form one replicate-level observation. For microbiota sequencing, cecal contents from one designated sampled hen per replicate were analyzed, so each microbiota sample represented one biological replicate rather than an average of the three sampled hens.

### 2.3. Sample Collection

On day 42, three clinically healthy hens with body weights close to the replicate mean were selected from each replicate after a 12 h feed withdrawal (water remained available), yielding 18 sampled hens per treatment and 72 hens in total. The identity of the replicate from which each hen originated was retained throughout sample collection, laboratory measurement, and statistical analysis. Approximately 10 mL of blood was collected from the wing vein of each hen. After standing at 4 °C for 30 min, blood was centrifuged at 3000 r/min for 10 min; serum was separated and stored at −80 °C. Sampled hens were euthanized by exsanguination. Duodenum, jejunum, and ileum segments were fixed in 4% paraformaldehyde for paraffin embedding and hematoxylin–eosin staining. Jejunal and ileal tissues were snap-frozen in liquid nitrogen and stored at −80 °C for intestinal barrier-related gene-expression analysis. Cecal contents were collected aseptically in sterile tubes, snap-frozen in liquid nitrogen, and stored at −80 °C for 16S ribosomal RNA (rRNA) gene amplicon sequencing.

### 2.4. Serum Lipid Analyses

Serum low-density lipoprotein cholesterol (LDL-C) and high-density lipoprotein cholesterol (HDL-C) concentrations were determined using commercial microplate assay kits (catalog nos. ADS-W-D012 and ADS-W-D011, respectively; Jiangsu Aidisheng Biological Technology Co., Ltd., Nanjing, China) according to the manufacturer’s instructions. Measurements from the three hens sampled within each replicate were averaged to obtain one replicate-level value for each serum index.

### 2.5. Intestinal Histomorphology

**Figure 1:**
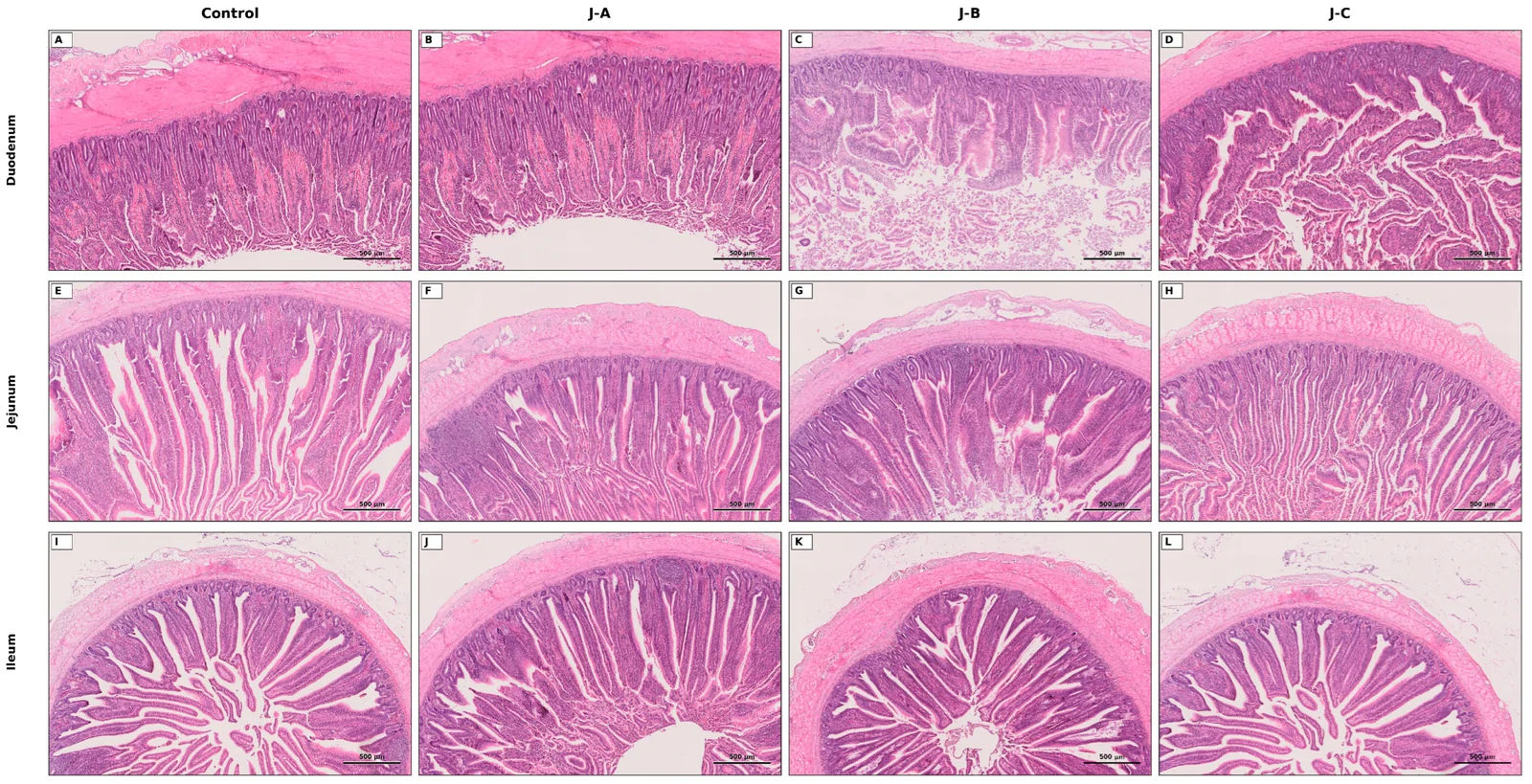
Representative hematoxylin-and-eosin-stained intestinal sections from Control, J-A, J-B, and J-C hens. Panels (**A**–**D**), duodenum; panels (**E**–**H**), jejunum; panels (**I**–**L**), ileum. Columns correspond to Control, J-A, J-B, and J-C, respectively. After sections with folding notes were excluded, the representative section for each treatment-segment combination was selected after quantification as the section closest to the group mean across the three standardized section-level indices (villus height, crypt depth, and their ratio). All panels show the same physical field width; scale bars = 500 μm. Control, basal diet without supplemental sterol-rich yeast hydrolysate (SYH); J-A, J-B, and J-C, basal diet supplemented with 0.2, 0.5, and 0.8 g SYH/kg diet, respectively. These images are representative visual examples and are not themselves evidence of statistical differences; statistical inference is based on the section-level measurements.

Fixed small-intestinal samples were paraffin embedded, sectioned at 3 μm, and stained with hematoxylin and eosin. The retained H&E service documentation records an Olympus DP26 microscope digital camera for image acquisition. The microscope or whole-slide-scanner body make and model were not recorded in the retained documentation and could not be retrieved. Duodenal, jejunal, and ileal morphology were examined using SlideViewer version 2.6.0.166179. One section from each of six biological samples per treatment and intestinal segment was evaluated. Within each section, villus height and crypt depth were measured in three intact, well-oriented, paired villus-crypt units that could be continuously traced from the villus tip to the crypt base; oblique, folded, torn, or incomplete units were excluded. Villus height was measured from the villus tip to the villus–crypt junction, and crypt depth from the same junction to the crypt base. The villus-height-to-crypt-depth ratio was calculated for each paired unit. The three measurements for villus height, crypt depth, and their ratio were averaged to generate one section-level value for each index. Individual villus–crypt units were treated as within-section repeated measurements rather than independent observations; thus, there were *n* = 6 section-level observations per treatment and intestinal segment. For Figure 1, after sections with folding notes were excluded, the representative section for each treatment–segment combination was selected as the section closest to the group mean across the three standardized section-level indices.

### 2.6. Digestive Enzyme Activities and Barrier-Related Gene Expression

Duodenal and ileal trypsin, chymotrypsin, lipase, and alpha-amylase activities were determined using commercial microplate assay kits (catalog nos. ADS-W-D004, ADS-W-D006, ADS-W-ZF005, and ADS-W-D032, respectively (Jiangsu Aidisheng Biological Technology Co., Ltd., Nanjing, China), according to the manufacturer’s instructions. Activities were expressed as nmol/min/g fresh weight (FW) for trypsin, chymotrypsin, and lipase and as U/g fresh weight for alpha-amylase. For each enzyme, the three hen-level measurements within a replicate were averaged before statistical analysis. For barrier-related gene expression, total ribonucleic acid (RNA) was extracted from jejunal mucosa using TRNzol Universal reagent (DP424; Tiangen Biotech, Beijing, China). RNA concentration and purity were measured with a NanoDrop 2000 spectrophotometer (Thermo Fisher Scientific, Waltham, MA, USA), and integrity was assessed by 1.5% agarose-gel electrophoresis. First-strand complementary deoxyribonucleic acid (cDNA) was synthesized using the RevertAid First Strand cDNA Synthesis Kit (K1622; Thermo Fisher Scientific). Quantitative real-time PCR was performed on an ABI 7500 Real-Time PCR System (Applied Biosystems, Waltham, MA, USA) using 2× SYBR Green qPCR Master Mix (B21203; Selleck Chemicals, Houston, TX, USA). The 20 μL reaction contained 100 ng cDNA, 0.5 μL each of forward and reverse primers, 10 μL of SYBR Green mix, 0.4 μL of 50× ROX reference dye, and RNase/DNase-free ddH_2_O. The program comprised 94 °C for 30 s; 40 cycles of 94 °C for 5 s and 60 °C for 30 s; and melting-curve analysis. Each biological sample was analyzed in technical triplicate, and technical-replicate cycle-threshold values were averaged before ΔCt calculation. qPCR was performed separately for each of the three sampled hens within a replicate; the three hen-level ΔCt values were then averaged to generate one replicate-level biological observation for statistical analysis. Beta-actin served as the reference gene, and relative expression of zonula occludens-1 (ZO-1), occludin, claudin-1, and mucin 2 (MUC2) was calculated using the 2^−ΔΔCt^ method. Primer sequences are presented in Table 1. Independent amplification-efficiency assessments for the target and reference assays could not be verified because standard-curve data were not available in the archived qPCR documentation. Replicate-level mean β-actin Ct values were therefore examined across treatments as a post hoc reference-gene check, but formal reference-gene stability validation using multiple candidate genes was not performed.

### 2.7. Cecal 16S rRNA Gene Amplicon Sequencing and Bioinformatics Analysis

Cecal-content samples from one designated hen per replicate were submitted to Novogene Co., Ltd. (Beijing, China), with six intended biological replicates per treatment. One J-A sample (J-A3) was damaged during transportation and did not meet the sequencing provider’s sample quality-control requirements; therefore, it was excluded from the final sequencing dataset, and 23 samples were retained (Control, n = 6; J-A, n = 5; J-B, n = 6; J-C, n = 6). DNA extraction was performed by the sequencing provider; however, the specific extraction-kit model and detailed extraction procedure were not recorded in the retained project-specific documentation and therefore could not be verified. DNA concentration and purity were assessed spectrophotometrically, and DNA integrity was evaluated by agarose gel electrophoresis. Only samples that met the sequencing provider’s quality-control criteria were used for polymerase chain reaction (PCR) amplification. The sequencing provider’s report documentation describes its amplicon PCR workflow as including ddH_2_O negative-control wells and states that downstream processing proceeds only when no band is observed in the PCR negative control. A negative extraction control was not separately documented in the retained project-specific files and therefore could not be verified.

The V3-V4 region of the bacterial 16S rRNA gene was amplified using primers 341F (5′-CCTAYGGGRBGCASCAG-3′) and 806R (5′-GGACTACNNGGGTATCTAAT-3′). Each PCR mixture contained 15 μL of Phusion High-Fidelity PCR Master Mix, 0.2 μM of each primer, and 10 ng of genomic DNA. Thermal cycling consisted of initial denaturation at 98 °C for 1 min; 30 cycles of 98 °C for 10 s, 50 °C for 30 s, and 72 °C for 30 s; and a final extension at 72 °C for 5 min. PCR products were purified using magnetic beads and pooled in equimolar amounts. Libraries were quantified using a Qubit fluorometer (Thermo Fisher Scientific, Waltham, MA, USA) and quantitative PCR and sequenced on an Illumina NovaSeq 6000 platform (Illumina, San Diego, CA, USA).

Raw paired-end reads were demultiplexed according to barcode sequences, and barcode and primer sequences were removed. Paired-end reads were merged using FLASH version 1.2.11 [30], residual reverse-primer sequences were removed using Cutadapt (version 3.3) [31], and read quality was filtered using fastp version 0.23.1 [32]. Reads containing more than five N bases were discarded; the quality threshold was Q19, and reads containing more than 15% low-quality bases were removed. Chimeric sequences were identified against the SILVA reference database and removed. Across the 23 retained samples, 2,430,612 raw paired-end reads were generated, 2,403,695 merged tags were obtained, 2,332,446 tags remained after quality filtering, and 1,849,697 Effective Tags remained after chimera removal. Denoising and amplicon sequence variant (ASV) inference were performed using the Divisive Amplicon Denoising Algorithm 2 (DADA2) [33] plugin in Quantitative Insights Into Microbial Ecology 2 (QIIME 2; release 2025.4) [34]. Taxonomy was assigned in QIIME 2 using the classify-sklearn method with a pretrained Naive Bayes classifier implemented in QIIME 2 (version 2025.4). against SILVA 138.2 [35]. The denoised feature table contained 1,590,610 reads and 5808 ASVs, with 46,001–87,267 reads per sample before rarefaction. Diversity analyses were rarefied to 46,001 reads per sample. Sample-level sequencing depth and observed ASV counts are provided in Supplementary Table S1.

Relative-abundance profiles were summarized at the phylum, family, and genus levels, and Venn diagrams, rank-abundance curves, species-accumulation boxplots, and rarefaction curves were generated in R version 4.0.3. Alpha diversity was assessed using Chao1, dominance, Good’s coverage, observed features, Pielou’s evenness, Shannon, and Simpson indices. Across the denoised reads, taxonomic assignment rates were 100.00% at kingdom, 98.53% at phylum, 98.53% at class, 98.14% at order, 88.66% at family, 76.25% at genus, and 66.45% at species level (Supplementary Table S2). Bray–Curtis dissimilarities were visualized by principal coordinate analysis (PCoA) using the ade4 package (https://cran.r-project.org/package=ade4, accessed on 21 August 2026) and ggplot2 package (https://cran.r-project.org/package=ggplot2, accessed on 21 August 2026) packages. Formal beta-diversity inference was evaluated by permutational multivariate analysis of variance (PERMANOVA) on the Bray–Curtis distance matrix, with 9999 label permutations [36]. Homogeneity of multivariate dispersion was assessed by PERMDISP in principal-coordinate space using group spatial medians, a small-sample bias adjustment for the slightly unequal group sizes, and 9999 residual permutations [37]. When the overall PERMANOVA was significant, pairwise PERMANOVA comparisons were examined and Holm adjustment was applied across the six treatment contrasts. Pairwise differential-abundance analysis at the family and genus levels was performed using metagenomeSeq [38]. *p* values were adjusted using the Benjamini–Hochberg false discovery rate, with q < 0.05 considered statistically significant. Differential-abundance results are presented as log-fold changes with 95% confidence intervals.

### 2.8. Statistical Analysis

Data were organized in Microsoft Excel and analyzed using IBM SPSS Statistics version 27.0. Each replicate was considered the experimental unit. For serum lipid indices and digestive enzyme activities, the three hen-level measurements within each replicate were averaged to generate one replicate-level observation; therefore, six independent observations were analyzed per treatment. For gene-expression outcomes, technical-replicate cycle-threshold values were averaged within each hen, ΔCt values were calculated for each hen, and the three hen-level ΔCt values within each replicate were averaged to generate one replicate-level biological observation; statistical inference was therefore based on six replicate-level observations per treatment. The 2^−ΔΔCt^ values are presented to facilitate biological interpretation. As a post hoc check of reference-gene behavior, replicate-level mean β-actin Ct values were compared among treatments by ordinary one-way ANOVA. For intestinal morphology, the three paired villus-crypt measurements within each section were averaged before analysis. The section-level mean associated with each replicate served as the observation (*n* = 6 per treatment and intestinal segment), and individual villus-crypt units were not treated as independent replicates. Residual normality and homogeneity of variance were assessed using the Shapiro–Wilk and Levene tests, respectively. Ordinary one-way analysis of variance (ANOVA) followed by Tukey-adjusted pairwise comparisons was used when assumptions were satisfied. Welch ANOVA followed by Games–Howell comparisons was used when residuals were approximately normal but variances were heterogeneous, and the Kruskal–Wallis test followed by Dunn comparisons with Holm adjustment was used when normality was not satisfied. Cecal microbiota alpha-diversity indices were analyzed using ordinary one-way ANOVA or the Kruskal–Wallis test according to the same criteria. Results are presented as treatment means. Pooled standard errors of the mean (SEM) are reported for all outcomes. For Welch ANOVA and Kruskal–Wallis outcomes, pooled SEM is provided as a descriptive summary on the original scale; inferential conclusions are based on the corresponding Welch or rank-based test and multiplicity-adjusted pairwise comparisons, where applicable. Because no single primary host endpoint was prespecified, host-trait findings were interpreted as exploratory, with emphasis placed on adjusted pairwise comparisons, consistency across related endpoints, and biological coherence rather than isolated *p* values. Statistical significance was set at *p* < 0.05.

For the exploratory microbiota–phenotype analysis, microbial and phenotype measurements were matched at the replicate level. Spearman correlation coefficients were calculated between the 30 displayed bacterial genera and 18 nonredundant host traits: six raw intestinal-morphology measurements, eight digestive-enzyme activities, and four barrier-related gene-expression outcomes. Villus-height-to-crypt-depth ratios were excluded from this analysis to avoid mathematical redundancy with their component measurements. The final matched dataset comprised 23 observations (Control, n = 6; J-A, n = 5; J-B, n = 6; J-C, n = 6) because the J-A3 microbiota sample was excluded from the final sequencing dataset. As a sensitivity analysis, rank-transformed microbial and phenotype variables were adjusted for treatment-group indicators before residual-rank correlations were calculated. Benjamini–Hochberg correction was applied across all 540 genus–phenotype tests. Nominal *p* values are displayed in the exploratory heatmap, whereas multiplicity-adjusted q values were used to judge statistical robustness.

## 3. Results

### 3.1. Serum Lipid Indices

Treatment affected low-density lipoprotein cholesterol (LDL-C; ordinary one-way ANOVA, *p* = 0.005; Table 2), whereas high-density lipoprotein cholesterol (HDL-C) did not differ among treatments (Welch ANOVA, *p* = 0.101). Tukey-adjusted comparisons showed that LDL-C was higher in J-C than in Control, J-A, and J-B (adjusted *p* = 0.032, 0.012, and 0.008, respectively), which did not differ from one another.

### 3.2. Intestinal Morphology

At the section level, overall treatment effects were detected for duodenal villus height (ordinary one-way ANOVA, *p* = 0.040), the duodenal villus-height-to-crypt-depth ratio (ordinary one-way ANOVA, *p* = 0.028), jejunal crypt depth (ordinary one-way ANOVA, *p* = 0.015), and ileal crypt depth (Kruskal–Wallis test, *p* = 0.035; Table 3). Tukey-adjusted comparisons showed that duodenal villus height was higher in J-A than in J-B (adjusted *p* = 0.025). The duodenal villus-height-to-crypt-depth ratio was higher in J-A than in the Control (adjusted *p* = 0.049) and J-B (adjusted *p* = 0.045), and jejunal crypt depth was higher in J-B than in the Control (adjusted *p* = 0.045). For ileal crypt depth, no Dunn pairwise comparison remained significant after Holm adjustment (all adjusted *p* ≥ 0.096). Duodenal crypt depth, jejunal villus height and villus-height-to-crypt-depth ratio, and ileal villus height and villus-height-to-crypt-depth ratio did not differ among treatments (*p* > 0.05). Representative hematoxylin-and-eosin-stained sections selected using the predefined objective rule are shown in Figure 1.

### 3.3. Intestinal Digestive Enzyme Activities

Duodenal lipase activity showed an overall treatment effect (Kruskal–Wallis, *p* = 0.047; Table 2), but no Dunn pairwise comparison remained significant after Holm adjustment (all adjusted *p* ≥ 0.086). Ileal lipase activity also differed overall (Welch ANOVA, *p* = 0.020); Games–Howell comparisons showed higher activity in J-C than in J-A (adjusted *p* = 0.020), whereas the other pairwise contrasts were not significant. Duodenal and ileal trypsin, chymotrypsin, and alpha-amylase activities did not differ among treatments (*p* > 0.05).

### 3.4. Intestinal Barrier-Related Gene Expression

Barrier-related transcript responses were endpoint-specific (Table 2). ZO-1 differed overall among treatments (Kruskal–Wallis, *p* = 0.008); Dunn–Holm comparisons showed higher expression in J-A than in the Control (adjusted *p* = 0.009) and J-B (adjusted *p* = 0.045), whereas J-C did not differ from the other groups. Occludin and claudin-1 also differed among treatments (both ordinary one-way ANOVA, *p* < 0.001), with J-A higher than Control, J-B, and J-C after Tukey adjustment. MUC2 differed overall (ordinary one-way ANOVA, *p* = 0.001); J-A and J-C were higher than the Control, whereas J-B did not differ significantly from any group. These transcript changes indicate modulation of barrier-related molecular markers, but they do not by themselves demonstrate improved intestinal barrier function; no tight-junction protein abundance, protein localization, or direct permeability endpoint was measured. As a post hoc reference-gene check, replicate-level mean β-actin Ct values did not differ among treatments (one-way ANOVA, *p* = 0.741); this comparison does not constitute formal multi-reference-gene stability validation.

### 3.5. Cecal Microbiota Alpha Diversity

No treatment effects were detected for Chao1 richness, dominance, Good’s coverage, observed features, Pielou’s evenness, Shannon diversity, or Simpson diversity (all *p* > 0.05; Table 4). Thus, within the available sample set, SYH did not significantly alter the assessed measures of cecal microbial richness, coverage, evenness, or alpha diversity. Given the limited microbiota sample size, these nonsignificant results indicate that no statistically robust alpha-diversity differences were detected under the present conditions; they should not be interpreted as proof that biological effects were absent.

### 3.6. Cecal Microbial Composition, Beta Diversity, and Differential Taxa

**Figure 2:**
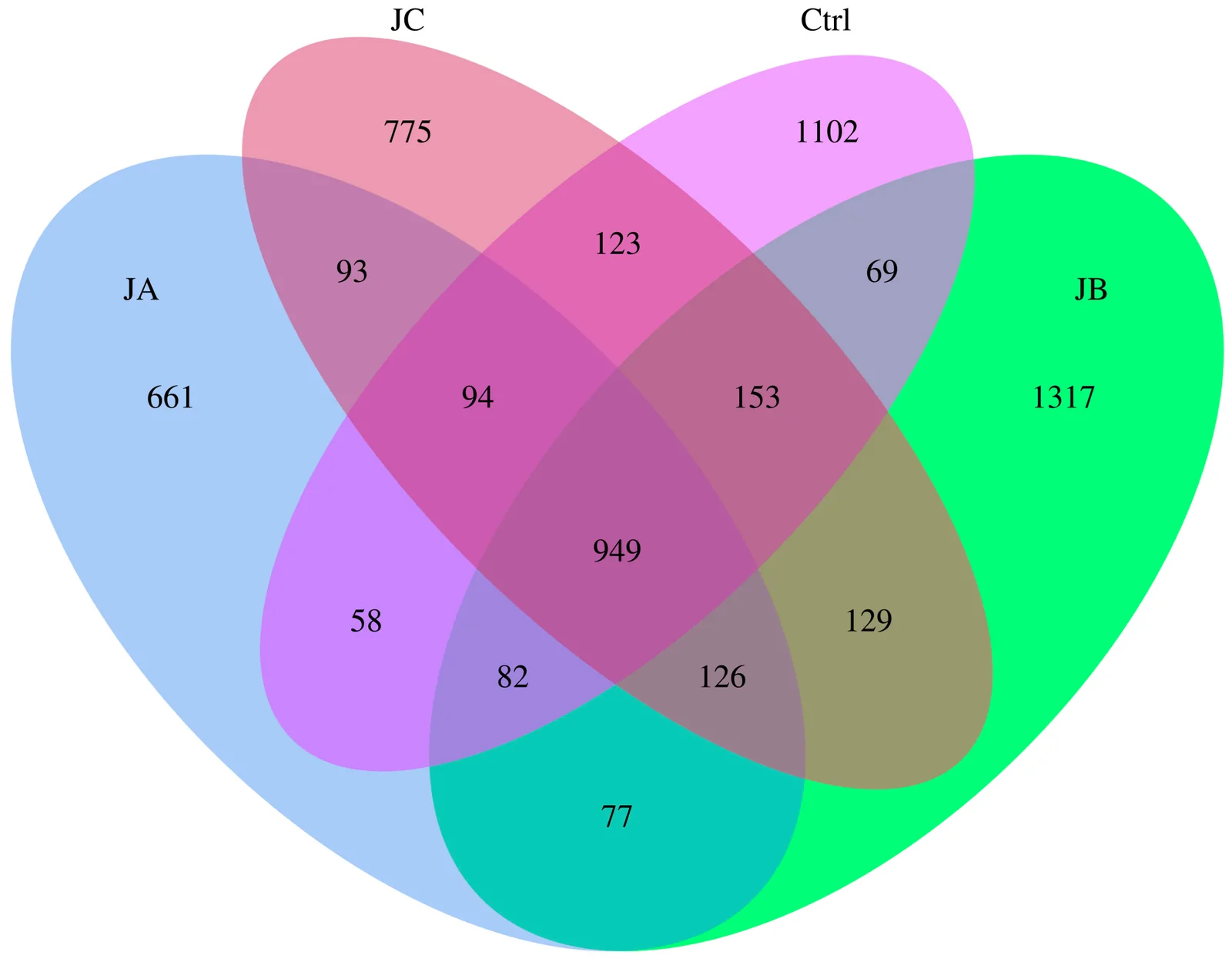
Venn diagram showing shared and unique cecal bacterial amplicon sequence variants (ASVs) among the four treatment groups. Numbers indicate ASV counts in each unique or overlapping region. Ctrl, basal diet without supplemental sterol-rich yeast hydrolysate (SYH); J-A, J-B, and J-C, basal diet supplemented with 0.2, 0.5, and 0.8 g SYH/kg diet, respectively.

Venn analysis identified 949 ASVs shared by all four treatments (Figure 2). The group-level unique-ASV counts were 1102, 661, 1317, and 775 for Control, J-A, J-B, and J-C, respectively. These counts are descriptive only and were not used to infer treatment effects on richness; neither Chao1 nor observed features differed among treatments.

**Figure 3:**
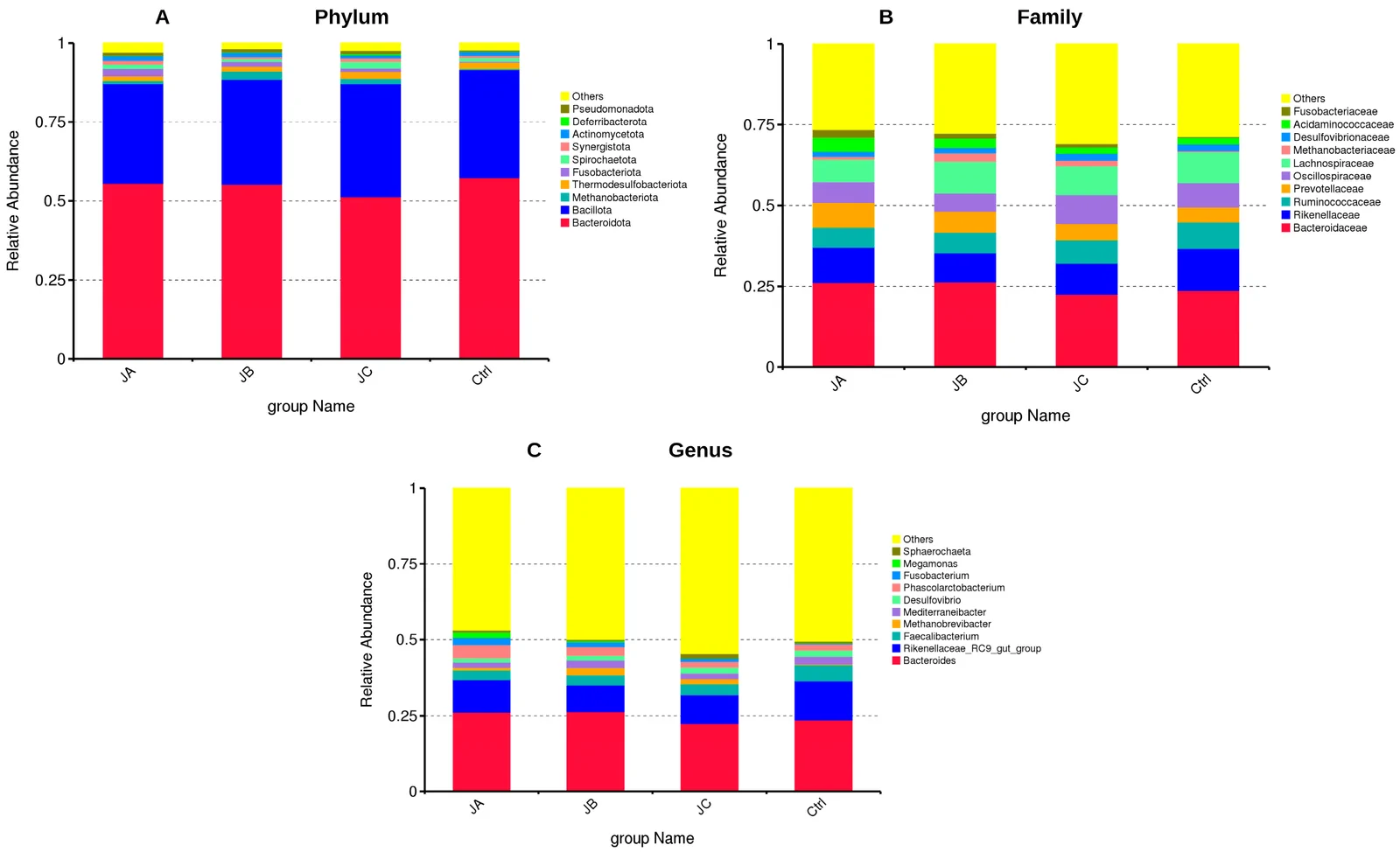
Relative-abundance profiles of the cecal bacterial community at the phylum (**A**), family (**B**), and genus (**C**) levels. Low-abundance taxa are combined as Others. Ctrl, basal diet without supplemental SYH; J-A, J-B, and J-C, basal diet supplemented with 0.2, 0.5, and 0.8 g SYH/kg diet, respectively.

At the phylum level, Bacteroidota and Bacillota predominated in all treatments, and the overall phylum-level profiles were broadly similar among groups. Bacteroidaceae, Rikenellaceae, Ruminococcaceae, Prevotellaceae, Oscillospiraceae, and Lachnospiraceae were among the major bacterial families. At the genus level, *Bacteroides* and Rikenellaceae_RC9_gut_group were the predominant annotated genera across treatments. These descriptive profiles indicate that the dominant cecal bacterial community was generally maintained following SYH supplementation (Figure 3).

**Figure 4:**
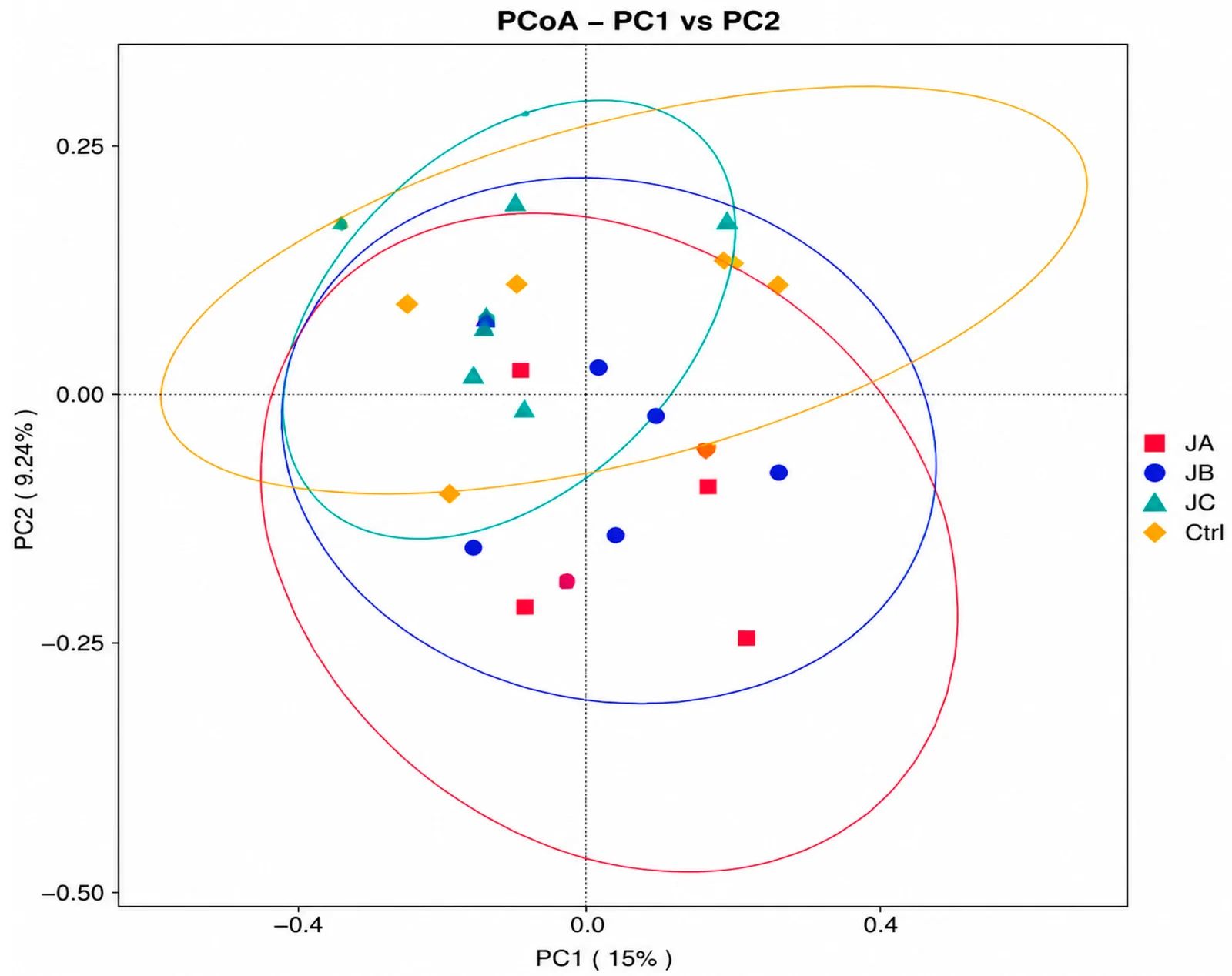
Principal coordinate analysis (PCoA) based on Bray–Curtis dissimilarities of cecal bacterial communities. PC1 and PC2 explained 15.00% and 9.24% of the total variation, respectively; ellipses indicate the distribution of samples within each treatment group. Ctrl, basal diet without supplemental SYH; J-A, J-B, and J-C, basal diet supplemented with 0.2, 0.5, and 0.8 g SYH/kg diet, respectively. Overall Bray–Curtis PERMANOVA: pseudo-F3,19 = 1.314, R^2^ = 0.172, *p* = 0.017; PERMDISP: F3,19 = 0.354, *p* = 0.776.

Principal coordinate analysis based on Bray–Curtis dissimilarities showed that PC1 and PC2 explained 15.00% and 9.24% of the total variation, respectively (Figure 4). Samples from the four treatments overlapped substantially in ordination space, with no visually discrete treatment clusters. Nevertheless, overall PERMANOVA detected a modest difference in community composition among treatments (pseudo-F3,19 = 1.314, R^2^ = 0.172, *p* = 0.017; 9999 permutations). PERMDISP did not detect heterogeneity of multivariate dispersion (F3,19 = 0.354, *p* = 0.776). In Holm-adjusted pairwise PERMANOVA, only J-A versus J-C remained significant (pseudo-F1,9 = 1.737, R^2^ = 0.162, adjusted *p* = 0.013).

**Figure 5:**
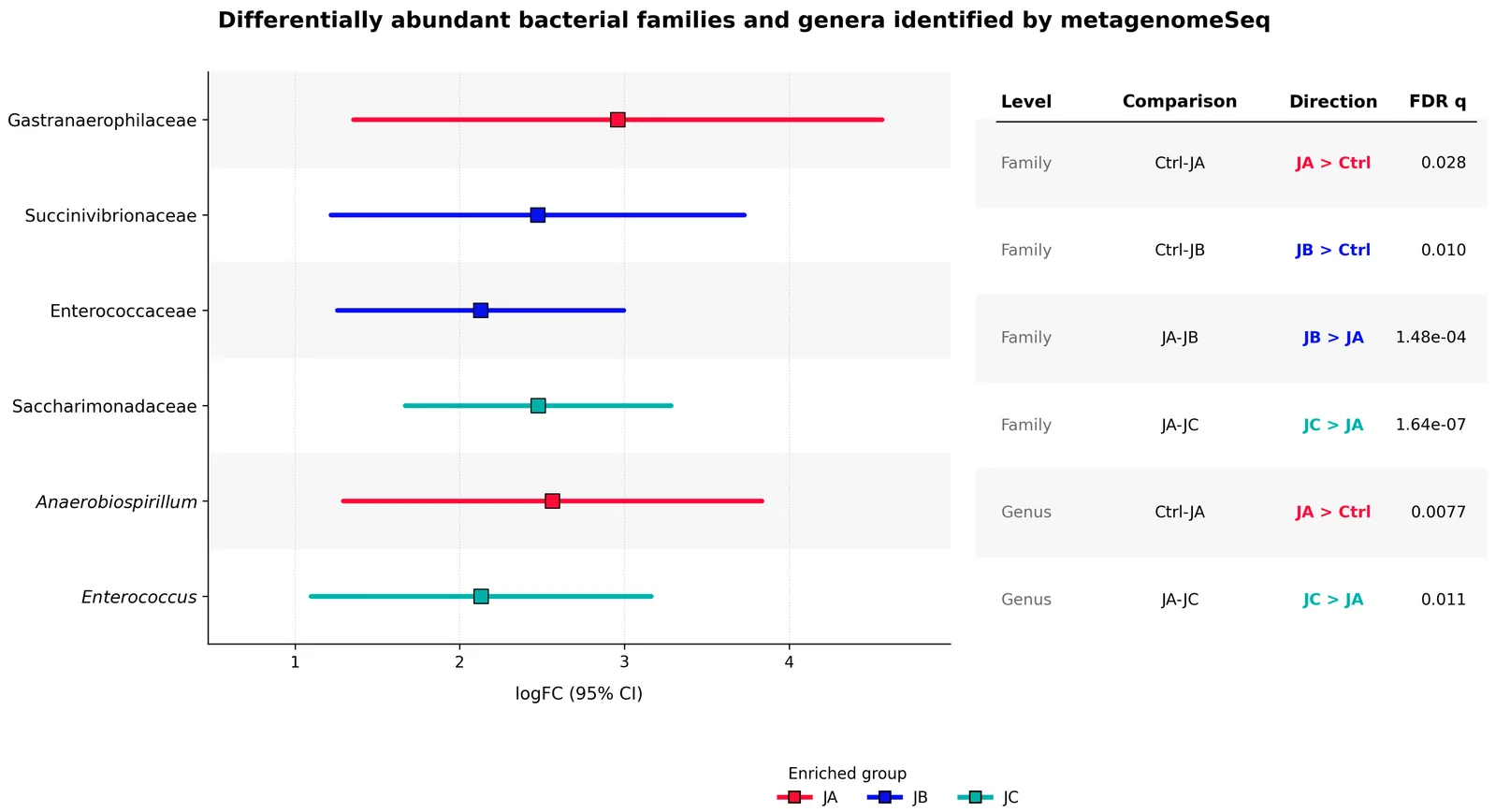
Differentially abundant bacterial families and genera identified using metagenomeSeq after Benjamini–Hochberg false-discovery-rate correction. Squares indicate estimated log fold changes, horizontal lines indicate 95% confidence intervals, and colors indicate the enriched treatment group. Only false-discovery-rate-significant taxa are shown. Ctrl, basal diet without supplemental SYH; J-A, J-B, and J-C, basal diet supplemented with 0.2, 0.5, and 0.8 g SYH/kg diet, respectively. The treatment contrast, direction of enrichment, and FDR-adjusted q value are shown for each displayed taxon.

Pairwise differential-abundance analysis using metagenomeSeq identified six bacterial taxa that differed after false-discovery-rate correction (Figure 5). At the family level, Gastranaerophilaceae was enriched in J-A compared with Control (*q* = 0.028), whereas Succinivibrionaceae was enriched in J-B compared with Control (*q* = 0.010). Enterococcaceae was enriched in J-B compared with J-A (*q* = 1.48 × 10^−4^), and Saccharimonadaceae was enriched in J-C compared with J-A (*q* = 1.64 × 10^−7^). At the genus level, *Anaerobiospirillum* was enriched in J-A compared with the Control (*q* = 0.0077), whereas *Enterococcus* was enriched in J-C compared with J-A (*q* = 0.011). The significant taxa differed across pairwise comparisons and did not exhibit a consistent monotonic response to increasing SYH supplementation.

### 3.7. Exploratory Microbiota–Phenotype Correlations

**Figure 6:**
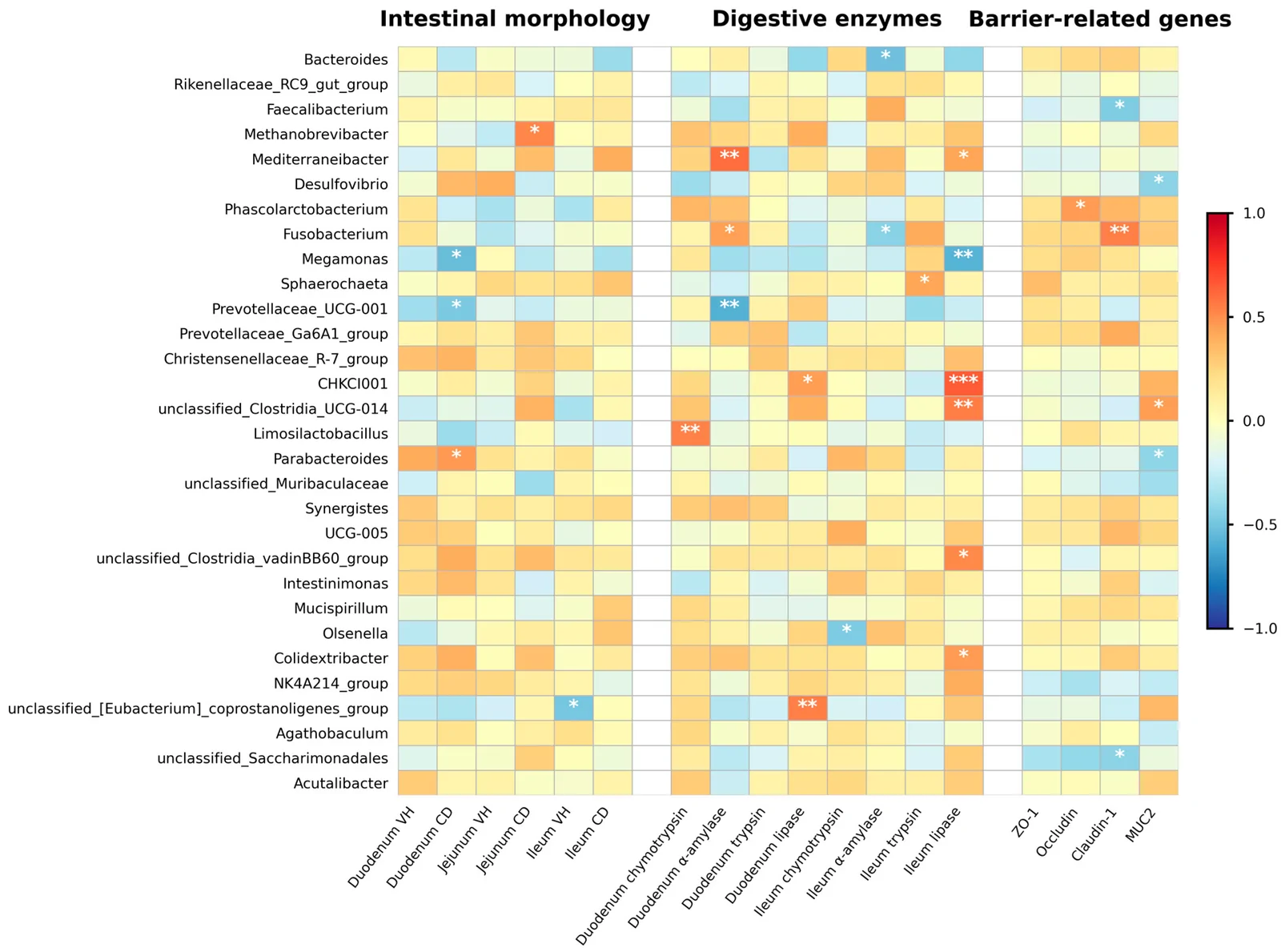
Exploratory Spearman correlation heatmap between the 30 displayed bacterial genera and 18 nonredundant host traits comprising intestinal morphology, digestive enzyme activities, and barrier-related gene expression. Colors represent Spearman correlation coefficients (ρ). * *p* < 0.05, ** *p* < 0.01, and *** *p* < 0.001 indicate nominal, unadjusted values; no association remained significant after Benjamini–Hochberg correction. The matched dataset comprised 23 replicate-level observations (Control, n = 6; J-A, n = 5; J-B, n = 6; J-C, n = 6). Control, basal diet without supplemental sterol-rich yeast hydrolysate (SYH); J-A, J-B, and J-C, basal diet supplemented with 0.2, 0.5, and 0.8 g SYH/kg diet, respectively.

None of the 540 genus–phenotype associations remained significant after Benjamini–Hochberg correction (all q > 0.05). At the nominal, unadjusted level, 28 associations had *p* < 0.05 (Figure 6). After recalculation of the intestinal-morphology block using the updated morphology dataset, five nominal associations were observed: Methanobrevibacter was positively associated with jejunal crypt depth (ρ = 0.518, *p* = 0.011), whereas Megamonas and Prevotellaceae_UCG-001 were negatively associated with duodenal crypt depth (ρ = −0.526, *p* = 0.010 and ρ = −0.476, *p* = 0.022, respectively). Parabacteroides was positively associated with duodenal crypt depth (ρ = 0.465, *p* = 0.026), and unclassified_[Eubacterium]_coprostanoligenes_group was negatively associated with ileal villus height (ρ = −0.486, *p* = 0.019). Among the unchanged digestive–enzyme correlations, relatively large nominal coefficients included a positive association between CHKCI001 and ileal lipase activity (ρ = 0.655, *p* < 0.001), a positive association between Mediterraneibacter and duodenal alpha-amylase activity (ρ = 0.596, *p* = 0.003), and negative associations of Prevotellaceae_UCG-001 with duodenal alpha-amylase activity (ρ = −0.580, *p* = 0.004) and Megamonas with ileal lipase activity (ρ = −0.575, *p* = 0.004).

The treatment-adjusted sensitivity analysis likewise yielded no multiplicity-corrected associations. Accordingly, the nominal correlations were treated as hypothesis-generating patterns and were not used to support causal or mechanistic conclusions.

## 4. Discussion

The principal finding was not a uniform improvement across outcomes but a selective pattern that differed by endpoint and inclusion level. J-C was distinguished by higher LDL-C than the other treatments and higher ileal lipase activity than J-A, whereas HDL-C and most digestive-enzyme activities did not differ. J-A showed the clearest increases in several barrier-related transcripts, although the pairwise pattern was not identical across ZO-1, occludin, claudin-1, and MUC2. Intestinal morphology changed only selectively, microbial alpha diversity remained stable, and the differential taxa were specific to individual pairwise comparisons. This pattern does not support a single coordinated improvement in intestinal health.

The serum response was restricted to LDL-C. J-C had higher LDL-C than Control, J-A, and J-B, whereas HDL-C did not differ among treatments after replicate-level reanalysis. Thus, the present data do not support a cholesterol-lowering or uniformly favorable lipoprotein response. Previous studies of yeast hydrolysates, yeast autolysates, and yeast extracts in laying hens have reported variable effects on egg traits and cholesterol-related outcomes [8,9,12,15,16,17,18,19], whereas phytosterols have often been investigated for hypolipidemic or hepatic effects in poultry [10,11]. The calculated sterol contributions in J-A, J-B, and J-C (6.2, 15.5, and 24.8 mg/kg diet) are in or below the lower portion of the 10–80 mg/kg phytosterol range tested in broilers and the 10–40 mg/kg phytosterol–ester range tested in laying hens [10,11]. These comparisons provide dose context only, because SYH is a multicomponent product and its sterol composition and bioavailability are not directly equivalent to purified phytosterol preparations. Total cholesterol, triglycerides, VLDL-related parameters, hepatic lipid metabolism, and yolk lipid deposition were not measured; therefore, HDL-C and LDL-C alone cannot establish improved lipid metabolism. Because lipid metabolism in laying hens is closely linked to hepatic synthesis and yolk deposition, the LDL-C difference should be interpreted together with egg-production, yolk-lipid, hepatic, and other lipid endpoints not included in the present analysis.

The morphological responses were selective and did not form a consistent pattern across intestinal segments. Duodenal villus height was higher in J-A than in J-B, while the duodenal villus-height-to-crypt-depth ratio was higher in J-A than in the Control and J-B. Jejunal crypt depth was higher in J-B than in the Control. Ileal crypt depth showed a significant overall treatment effect, but no Dunn pairwise comparison remained significant after Holm adjustment; accordingly, this result does not identify a specific pair of groups as different. The remaining morphological indices did not differ among treatments. These findings do not support a generalized improvement in intestinal architecture. Previous poultry studies have reported changes in villus height, crypt depth, or related morphological indices after supplementation with yeast-derived products, but the direction and magnitude of response vary with product type, inclusion level, age, challenge status, and experimental context [1,2,7,14,20,21]. Accordingly, the present histomorphological findings are interpreted as segment- and endpoint-specific responses; morphology alone does not demonstrate increased nutrient absorption or barrier function.

Digestive-enzyme responses were limited and segment-specific. Duodenal lipase showed a significant overall treatment effect, but no Dunn–Holm pairwise contrast remained significant. Ileal lipase was higher in J-C than in J-A, whereas the remaining ileal lipase comparisons and all trypsin, chymotrypsin, and alpha-amylase comparisons were nonsignificant. Previous work has linked yeast culture or yeast-cell-wall supplementation with altered digestive enzyme activities in poultry [3,6,14,20,21]. The present results therefore provide only limited evidence of a selective lipase response and do not identify the responsible SYH component. Accordingly, these findings are described as alterations in tissue-associated enzyme activity; they do not establish increased luminal secretion, improved nutrient digestibility, feed efficiency, or production performance.

Barrier-related transcripts showed selective and non-monotonic responses. J-A had higher ZO-1 than the Control and J-B, while J-C was intermediate and did not differ from the other groups. Occludin and claudin-1 were higher in J-A than in the Control, J-B, and J-C. MUC2 was higher in J-A and J-C than in the Control, with J-B intermediate and not significantly different from any group. Reports on yeast hydrolysates, nucleotides, and cell-wall products support the possibility that yeast-derived fractions influence tight-junction and mucosal responses [1,4,5,7,16,17]. The observed pattern is non-monotonic; however, the present experiment was not designed or statistically powered to establish a nonlinear dose–response curve, threshold, or hormetic mechanism. These transcript responses may reflect the modulation of molecular components associated with junctional and mucosal barrier integrity; however, messenger RNA abundance does not confirm junctional protein abundance, localization, or functional permeability. Future work should combine transcript measurements with protein abundance/localization and direct permeability assays.

The microbiota results also support a selective rather than global response. None of the alpha-diversity indices differed among treatments, and Bacteroidota, Bacillota, Bacteroides, and Rikenellaceae_RC9_gut_group remained dominant across groups, consistent with the diverse fermentative community typically reported in the chicken cecum [22,23,24,25,39]. Although samples overlapped substantially in the Bray–Curtis PCoA, PERMANOVA detected a modest overall treatment-associated difference in community composition (R^2^ = 0.172, *p* = 0.017), while the spatial-median PERMDISP with small-sample bias adjustment was nonsignificant (*p* = 0.776). Only J-A versus J-C remained significant after Holm adjustment in the pairwise PERMANOVA. Thus, visual overlap in ordination space should not be equated with statistical equality, but the effect size, limited sample size, and restricted pairwise support warrant cautious interpretation. Likewise, nonsignificant alpha-diversity results do not demonstrate absence of biological effects; they indicate that no statistically robust differences in the assessed alpha-diversity metrics were detected under the present experimental conditions and sample size.

After false-discovery-rate correction, metagenomeSeq identified six differentially abundant taxa, but the comparisons did not converge on a common supplementation response. Gastranaerophilaceae and Anaerobiospirillum differed between J-A and Control, Succinivibrionaceae differed between J-B and Control, and the remaining differences involved J-A as the reference group rather than the unsupplemented Control. Accordingly, a difference between J-B or J-C and J-A should not be interpreted as evidence that the same taxon differed from Control. The dispersion of significant findings across pairwise contrasts, together with the absence of a monotonic dose pattern, indicates limited, taxon-specific ecological shifts rather than uniform microbial modulation by SYH.

Functional interpretation of these taxa remains constrained. Gastranaerophilaceae and Saccharimonadaceae were resolved only at the family level, and increased Succinivibrionaceae or Anaerobiospirillum cannot demonstrate altered carbohydrate fermentation without functional genes or fermentation metabolites. Similarly, the greater Enterococcus abundance in J-C than in J-A cannot be classified as beneficial or harmful because V3-V4 16S rRNA sequencing does not reliably resolve species or strains [40]. Differential-abundance findings are also sensitive to analytical method and preprocessing [41]. Consequently, the identified taxa should be viewed as candidates for independent validation rather than mechanistic mediators of the host responses.

The correlation analysis did not provide robust evidence linking bacterial genera to host traits. Although 28 of 540 tests were nominally significant, approximately 27 would be expected by chance at an unadjusted threshold of *p* < 0.05, and none survived Benjamini–Hochberg correction. The treatment-adjusted sensitivity analysis reached the same conclusion. These results are therefore consistent with chance-level enrichment of nominal associations rather than a stable microbiota–phenotype network. The limited number of matched replicate-level observations further reduced power, and shared treatment effects could influence both microbial abundance and host traits. The correlations should be used only to prioritize hypotheses for larger studies. Accordingly, failure to detect FDR-significant correlations should not be interpreted as evidence that biological relationships are absent; the analysis had limited power and remains hypothesis-generating.

Across endpoint families, the responses were dispersed rather than concentrated at a single inclusion level. J-C was characterized by higher LDL-C and higher ileal lipase than J-A, while J-A showed the clearest increases in several barrier-related transcripts; MUC2 was elevated relative to Control in both J-A and J-C. Neither pattern was accompanied by consistent morphological improvement or broad microbial restructuring. This divergence explains why the present data do not support selecting one inclusion level as optimal and cautions against interpreting isolated significant outcomes as evidence of overall efficacy. Similar context-dependent responses have been reported for yeast-derived additives in laying and breeder hens [8,9,12,15,16,17,18,19].

Several limitations should be acknowledged. The study was conducted in a single genotype of broiler breeder hens at a specific age and the late-laying production stage over a relatively short 6-week period; therefore, generalization to other genotypes, ages, production stages, and longer-term conditions should be made cautiously. Barrier-related messenger RNA was measured without protein abundance, localization, or direct permeability testing; enzyme activities were not accompanied by nutrient digestibility; production and egg-quality endpoints were outside the scope of the present analysis, while yolk-lipid and hepatic endpoints were not assessed here. In addition, total cholesterol, triglycerides, and very-low-density-lipoprotein-related parameters were not measured, limiting a comprehensive assessment of the serum lipid response. Standard-curve data were not available in the archived qPCR documentation, so target/reference amplification efficiencies could not be independently verified. Although replicate-level β-actin Ct values showed no detectable treatment-associated difference in the post hoc comparison, formal reference-gene stability validation using multiple candidate genes was not performed. Product-specific assay detection ranges and intra- and inter-assay coefficients of variation were not retained in the archived assay records. One microbiota sample was excluded after transport damage, leaving 23 observations; this sample size limited statistical power, particularly for alpha-diversity and correlation analyses. Microbial analyses were based on relative abundance rather than absolute loads or direct functional measurements. The specific DNA extraction-kit model, detailed extraction procedure, and use of a negative extraction control could not be verified from the retained project-specific sequencing documentation; the provider report documentation describes the PCR negative-control practice used in its amplicon workflow. These documentation gaps limit full methodological reproducibility. The correlation analysis was restricted to 30 displayed genera and 18 host traits. Finally, SYH contained sterols, mannans, nucleotides, amino nitrogen, and other fractions, so the observed responses cannot be attributed specifically to sterols. These limitations constrain mechanistic interpretation and generalization beyond the tested conditions.

Future studies should prespecify primary outcomes and use designs that link production, egg and hepatic lipid traits, direct barrier measurements, nutrient digestibility, microbial absolute abundance, and fermentation metabolites at the same replicate level. Full functional profiling and independent validation of the differential taxa would help determine whether the low- and high-inclusion response patterns are reproducible and whether either pattern has practical value. Functional microbiome measurements, such as metagenomic or metatranscriptomic profiling together with fermentation metabolites, would be needed to test microbial mechanisms directly.

From a practical perspective, the present findings do not support recommending any one of the tested SYH inclusion levels as universally optimal for poultry production. Because the present analysis focused on serum lipid indices, intestinal traits, and cecal microbiota, it does not by itself establish whether any of the tested inclusion levels confers a consistent production advantage. Further validation integrating production and health endpoints under commercial conditions is therefore needed before a routine inclusion level can be recommended.

## 5. Conclusions

Under the conditions of this 6-week study, dietary SYH produced selective, inclusion-level- and trait-specific responses in yellow-feathered broiler breeder hens. LDL-C was higher in J-C than in the other treatments, whereas HDL-C did not differ. Duodenal lipase showed an overall treatment effect without a significant adjusted pairwise contrast, and ileal lipase was higher in J-C than in J-A. Barrier-related transcripts also varied selectively: J-A showed higher ZO-1 than the Control and J-B and higher occludin and claudin-1 than the other groups, while MUC2 was higher in J-A and J-C than in the Control. The enzyme-activity findings should not be interpreted as direct evidence of improved nutrient digestion, and the transcript responses indicate modulation of barrier-related molecular markers but do not by themselves establish improved intestinal barrier integrity. Morphological responses were selective and did not show a consistent pattern across intestinal segments. Microbial alpha diversity did not differ, whereas Bray–Curtis PERMANOVA indicated a modest overall difference in community composition; pairwise support was limited, and the six FDR-significant taxa remained specific to individual treatment contrasts without a monotonic dose pattern. No microbiota–phenotype association remained significant after multiple-testing correction. Therefore, the current endpoints do not identify a single optimal inclusion level or demonstrate a broad improvement in intestinal health, and the responses cannot be attributed specifically to the sterol fraction of this multicomponent product.

## Figures and Tables

**Table 1:** Genes and primer sequences.

Gene	Orientation	Primer Sequence (5′-3′)	Product Size (bp)
β-actin	F	CTTCACCACCACAGCCGAGAGAG	222
	R	CCACAGGACTCCATACCCAAGAAAG	
ZO-1	F	TCTCCCTAAAGGCGAAGAAGTAACC	182
	R	ATAGCGTGTCCACAACCCGAAAC	
Occludin	F	TGTGGGTTCCTCATCGTCATCC	208
	R	TCTCCGAGTAGGCAAGCGTGG	
Claudin-1	F	GATGCGGATGGCTGTCTTTGGT	150
	R	GGCTGGGTGGGTAGGATGTTTC	
MUC2	F	GAGCCACCTCCTAAACCCACCTG	93
	R	GCAATCACACTCCCAATGCCAAC	

Note: F, forward; R, reverse; bp, base pairs; ZO-1, zonula occludens-1; MUC2, mucin 2.

**Table 2:** Effects of dietary sterol-rich yeast hydrolysate on serum lipid indices, duodenal and ileal digestive enzyme activities, and jejunal barrier-related gene expression.

Item	Control	J-A	J-B	J-C	Pooled SEM	*p*-Value
**Serum lipid indices**						
HDL-C (mmol/L) ^‡^	0.66	0.94	0.58	1.37	0.19	0.101
LDL-C (mmol/L)	0.48 ^b^	0.42 ^b^	0.40 ^b^	0.86 ^a^	0.09	0.005
**Duodenal digestive enzyme activities**						
Trypsin (nmol/min/g FW)	151.32	169.01	138.92	151.78	12.05	0.391
Chymotrypsin (nmol/min/g FW) ^†^	166.27	191.86	249.52	210.51	40.98	0.819
Lipase (nmol/min/g FW) ^†^	547.03	540.84	596.24	682.18	38.75	0.047
α-Amylase (U/g FW) ^†^	401.43	996.77	738.54	1200.64	474.63	0.829
**Ileal digestive enzyme activities**						
Trypsin (nmol/min/g FW)	184.72	204.59	193.59	199.93	11.98	0.677
Chymotrypsin (nmol/min/g FW) ^†^	150.64	148.00	148.22	153.18	7.13	0.962
Lipase (nmol/min/g FW) ^‡^	453.84 ^ab^	388.83 ^b^	452.99 ^ab^	588.38 ^a^	29.05	0.020
α-Amylase (U/g FW) ^†^	324.66	154.11	152.22	198.07	90.49	0.713
**Jejunal barrier-related gene expression**						
ZO-1 ^†^	1.02 ^b^	2.15 ^a^	1.10 ^b^	1.34 ^ab^	0.27	0.008
Occludin	1.03 ^b^	3.94 ^a^	1.44 ^b^	1.55 ^b^	0.39	<0.001
Claudin-1	1.02 ^b^	3.72 ^a^	1.23 ^b^	1.41 ^b^	0.47	<0.001
MUC2	1.03 ^b^	1.89 ^a^	1.49 ^ab^	2.25 ^a^	0.21	0.001

Note: Control, basal diet without supplemental SYH; J-A, J-B, and J-C, basal diet supplemented with 0.2, 0.5, and 0.8 g SYH/kg diet, respectively. HDL-C, high-density lipoprotein cholesterol; LDL-C, low-density lipoprotein cholesterol; FW, fresh weight; ZO-1, zonula occludens-1; MUC2, mucin 2; SEM, standard error of the mean. Three hens were sampled from each of six replicates per treatment, and their measurements were averaged to obtain one replicate-level observation; thus, there were n = 6 replicates per treatment. For gene-expression outcomes, technical-replicate Ct values were averaged first, inference was based on replicate-level ΔCt values, and replicate-level 2^−ΔΔCt^ values are displayed for biological interpretation. Values are treatment means. Pooled SEM is reported for all outcomes as a descriptive summary. ^†^, Kruskal–Wallis test; ^‡^, Welch ANOVA; unmarked outcomes, ordinary one-way ANOVA. When the overall test was significant, ^†^, ^‡^, and unmarked outcomes were followed by Dunn comparisons with Holm adjustment, Games–Howell comparisons, and Tukey-adjusted comparisons, respectively. For ^†^ and ^‡^ outcomes, pooled SEM is descriptive on the original scale and was not used for inferential testing. For duodenal lipase ^†^, the overall Kruskal–Wallis test was significant (*p* = 0.047), but no Dunn–Holm pairwise comparison remained significant (all adjusted *p* ≥ 0.086). Within a row, means without a common lowercase superscript differ after the corresponding multiplicity-adjusted pairwise comparison (*p* < 0.05).

**Table 3:** Intestinal morphological indices among treatment groups.

Item	Control	J-A	J-B	J-C	Pooled SEM	*p*-Value
**Duodenum**						
Villus height (μm)	1018.73 ^ab^	1119.46 ^a^	884.92 ^b^	1036.58 ^ab^	53.07	0.040
Crypt depth (μm)	209.64	181.57	183.42	207.88	12.25	0.235
Villus height:crypt depth	4.91 ^b^	6.26 ^a^	4.89 ^b^	5.05 ^ab^	0.34	0.028
**Jejunum**						
Villus height (μm)	1062.84	918.57	905.42	994.36	81.65	0.508
Crypt depth (μm)	176.48 ^b^	178.92 ^ab^	204.63 ^a^	201.47 ^ab^	6.98	0.015
Villus height:crypt depth	6.06	5.16	4.44	4.96	0.45	0.115
**Ileum**						
Villus height (μm)	899.82	861.47	732.16	896.35	69.40	0.306
Crypt depth (μm) ^†^	166.84	188.73	169.21	191.56	5.72	0.035
Villus height:crypt depth	5.42	4.59	4.35	4.70	0.42	0.339

Note: Control, basal diet without supplemental SYH; J-A, J-B, and J-C, basal diet supplemented with 0.2, 0.5, and 0.8 g SYH/kg diet, respectively. n = 6 section-level observations per treatment and intestinal segment. For each section, villus height and crypt depth were measured in three intact, well-oriented, paired villus–crypt units, and the three measurements for each index were averaged before statistical analysis; individual units were not treated as independent observations. SEM, standard error of the mean; MSwithin, within-group mean square. Pooled SEM = √(MSwithin/6) for all rows and is used as a descriptive summary. ^†^, Kruskal–Wallis test; unmarked outcomes, ordinary one-way ANOVA. When the overall test was significant, ^†^ and unmarked outcomes were followed by Dunn comparisons with Holm adjustment and Tukey-adjusted comparisons, respectively. For the ^†^ outcome, treatment means and pooled SEM are descriptive on the original scale and were not used for inferential testing. Ileal crypt depth ^†^ showed a significant overall Kruskal–Wallis result (*p* = 0.035), but no Dunn–Holm pairwise comparison remained significant (all adjusted *p* ≥ 0.096). Within a row, means without a common lowercase superscript differ after multiplicity-adjusted pairwise comparison (*p* < 0.05).

**Table 4:** Cecal microbiota alpha-diversity indices among treatment groups.

Item	Control	J-A	J-B	J-C	Pooled SEM	*p*-Value
Chao1 index	872.59	890.32	966.39	989.76	57.50	0.412
Dominance index	0.0110	0.0100	0.0092	0.0087	0.0007	0.133
Good’s coverage ^†^	0.9992	0.9990	0.9987	0.9990	0.0002	0.300
Observed features	859.67	858.80	933.17	964.33	56.23	0.459
Pielou’s evenness index	0.8070	0.8048	0.8135	0.8172	0.0042	0.180
Shannon index	7.86	7.84	8.00	8.10	0.10	0.230
Simpson index	0.9890	0.9900	0.9908	0.9913	0.0007	0.133

Note: Control, J-B, and J-C, n = 6; J-A, n = 5. SEM, standard error of the mean. Pooled SEM under unequal sample sizes was calculated using the harmonic mean sample size (n_h_ = 5.714) for all rows and is presented as a descriptive summary. ^†^, Kruskal–Wallis test; unmarked outcomes, ordinary one-way ANOVA. For the ^†^ outcome, treatment means and pooled SEM are descriptive on the original scale and were not used for inferential testing; inference was rank-based. Control, basal diet without supplemental sterol-rich yeast hydrolysate (SYH); J-A, J-B, and J-C, basal diet supplemented with 0.2, 0.5, and 0.8 g SYH/kg diet, respectively.

## Data Availability

The processed microbiota results and additional sequencing quality-control summaries are included in the article and Supplementary Materials. The authors retain the complete raw and processed 16S rRNA gene amplicon sequencing dataset supplied by the sequencing provider. The complete raw sequencing data and analysis outputs are available from the corresponding author upon reasonable request.

## Supplementary Materials

The following supporting information can be downloaded at: https://www.mdpi.com/article/10.3390/ani16162620/s1, Table S1: Sample-level 16S rRNA gene amplicon sequencing depth and observed ASV counts; Table S2: Taxonomic assignment rates across denoised reads.

## Abbreviations

ANOVA, analysis of variance; ASV, amplicon sequence variant; cDNA, complementary deoxyribonucleic acid; CI, confidence interval; DADA2, Divisive Amplicon Denoising Algorithm 2; ddH_2_O, double-distilled water; dNTP, deoxynucleotide triphosphate; FDR, false discovery rate; FW, fresh weight; HDL-C, high-density lipoprotein cholesterol; LDL-C, low-density lipoprotein cholesterol; M-MuLV, Moloney murine leukemia virus; MUC2, mucin 2; PERMANOVA, permutational multivariate analysis of variance; PERMDISP, permutational analysis of multivariate dispersions; PCoA, principal coordinate analysis; QIIME, Quantitative Insights Into Microbial Ecology; RNA, ribonucleic acid; ROX, 6-carboxy-X-rhodamine; rRNA, ribosomal RNA; SEM, standard error of the mean; SYH, sterol-rich yeast hydrolysate; ZO-1, zonula occludens-1.

## Appendix A

**Table A1:** Composition and nutrient levels of the basal diet.

Item	Content
**Ingredients (%)**	
Corn	61.55
Soybean meal	25.50
Soybean oil	1.80
Limestone	9.00
Dicalcium phosphate	1.20
DL-Methionine	0.17
Sodium chloride	0.22
Choline chloride	0.11
Sodium bicarbonate	0.17
Premix ^1^	0.28
Total	100.00
**Nutrient levels**	
Crude fat (%)	3.43
Crude protein (%)	14.78
Available phosphorus (%) ^2^	0.37
Calcium (%)	2.93
Metabolizable energy (MJ/kg) ^2^	12.92

Note: ^1^ The premix provided per kilogram of diet: vitamin A, 10,000 IU; vitamin D3, 3500 IU; vitamin E, 28 IU; vitamin K3, 2.2 mg; vitamin B1, 2.5 mg; vitamin B2, 3.5 mg; vitamin B6, 3.0 mg; vitamin B12, 0.025 mg; biotin, 0.25 mg; folic acid, 0.8 mg; niacin, 32.0 mg; calcium D-pantothenate, 12.0 mg; Fe, 90 mg; Cu, 18 mg; Mn, 90 mg; Zn, 70 mg; Se, 0.3 mg; and I, 0.5 mg. ^2^ Metabolizable energy and available phosphorus were calculated values; crude fat, crude protein, and calcium were analyzed values.
