# Supplementary material for: Effects of Dietary Sterol-Rich Yeast Hydrolysate on Serum Lipid Indices, Intestinal Morphology, Digestive Enzyme Activities, Barrier-Related Gene Expression, and Cecal Microbiota in Yellow-Feathered Broiler Breeder Hens
Source: Animals (Basel). 2026 Aug 21;16(16):2620. doi: 10.3390/ani16162620 (PMC13509503; doi:10.3390/ani16162620)
Supplement: Supplementary file 1 [file animals-16-02620-s001.zip › animals-4495598-supplementary.pdf]

Supplementary Materials

Effects of Dietary Sterol-Rich Yeast Hydrolysate on Serum Lipid Indices, Intestinal Morphology, Digestive Enzyme Activities, Barrier-Related Gene Expression, and Cecal Microbiota in Yellow-Feathered Broiler Breeder Hens

Table S1. Sample-level 16S rRNA gene amplicon sequencing depth and observed ASV counts.

Sample	Treatment	Raw PE reads	Merged tags	Qualified tags	Effective tags	Denoised reads	Observed ASVs
JA1	J-A	105,154	103,887	101,103	81,405	71,727	932
JA2	J-A	106,379	104,748	101,499	85,871	75,860	783
JA4	J-A	105,148	103,642	100,140	83,661	74,014	842
JA5	J-A	106,549	105,358	102,360	78,804	67,152	927
JA6	J-A	105,086	103,623	100,596	81,653	70,003	810
JB1	J-B	113,785	112,893	110,222	57,396	46,001	1,360
JB2	J-B	104,132	102,615	99,434	76,045	63,884	739
JB3	J-B	104,655	103,462	100,334	82,763	72,200	805
JB4	J-B	103,438	102,280	99,098	83,409	73,512	814
JB5	J-B	106,048	104,719	101,488	82,159	69,265	947
JB6	J-B	102,140	100,873	97,650	80,906	70,457	934
JC1	J-C	102,335	101,238	98,156	83,852	73,319	840
JC2	J-C	108,606	107,279	104,410	93,158	83,324	1,069
JC3	J-C	119,591	118,248	114,731	100,006	87,267	1,075
JC4	J-C	103,976	102,573	99,252	81,435	71,175	969
JC5	J-C	104,100	102,621	99,566	80,174	67,714	897
JC6	J-C	103,733	102,585	99,642	90,059	80,055	936
Ctrl1	Control	103,578	102,765	99,540	77,742	65,105	839
Ctrl2	Control	103,299	102,492	99,509	78,690	65,308	924
Ctrl3	Control	104,056	103,315	100,420	70,887	57,569	778
Ctrl4	Control	106,733	105,966	102,942	73,992	62,066	974
Ctrl5	Control	104,532	103,743	100,627	73,410	62,407	860
Ctrl6	Control	103,559	102,770	99,727	72,220	61,226	783

Note: Raw PE reads are the original paired-end read counts; merged tags are reads after paired-end merging; qualified tags are retained after quality filtering; Effective tags are retained after chimera removal; denoised reads and observed ASVs are from the final DADA2/QIIME 2 feature table before rarefaction. J-A3 was not included because the sample was damaged during transportation and failed the provider's sample quality-control requirements.

Table S2. Taxonomic assignment rates across denoised reads.

Taxonomic level	Assigned reads	Assignment rate (%)
Kingdom	1,590,604	100.00
Phylum	1,567,216	98.53
Class	1,567,208	98.53
Order	1,560,951	98.14
Family	1,410,158	88.66
Genus	1,212,808	76.25
Species	1,057,012	66.45

Note: Assignment rates were calculated relative to the 1,590,610 reads in the final denoised feature table. The kingdom-level value rounds to 100.00%.
